# Accelerated Storage Induced Structural Evolution in Natural Rubber: A Comparative Study of Two Constant Viscosity Treatment Methods

**DOI:** 10.3390/polym17070960

**Published:** 2025-04-01

**Authors:** Danhua Yun, Wenfeng Peng, Hongtu Lin, Jianhe Liao, Lusheng Liao

**Affiliations:** 1 School of Materials Science and Engineering, Hainan University, Haikou 570228, China; 22210805000014@hainanu.edu.cn; 2 Hainan Provincial Key Laboratory of Natural Rubber Processing, Agricultural Products Processing Research Institute, Chinese Academy of Tropical Agricultural Sciences, Zhanjiang 524001, China; pwfpwx@163.com (W.P.); zjoulht@126.com (H.L.)

**Keywords:** constant viscosity natural rubber, accelerated storage, dry-mixing method, latex-mixing method

## Abstract

In order to compare the effects of current mainstream preparation methods for the constant viscosity natural rubber (CV) on structure during storage, this study used dry-mixing and latex-mixing methods to prepare the CV. The variation in mesostructure and microstructure of the CV prepared by the two constant viscosity treatment methods after accelerated storage for 48 h was analyzed. The result shows that both methods for preparing the CV can keep the macrogel content almost consistent after accelerated storage, while the microgel_>1μ_ content increased slightly. Meanwhile, both methods for preparing the CV can also stably maintain the molecular weight, the molecular weight distribution, and the shape of molecular chains after accelerated storage. However, the CV prepared by dry-mixing method demonstrated superior constant viscosity performance in Mooney viscosity (ML) and Wallace plasticity (P_0_) during accelerated storage. The latex-mixing method for preparing the CV showed better advantage in preserving the number of branches per chain during accelerated storage.

## 1. Introduction

Natural rubber (NR), a polymeric material, is made of natural latex by solidification, drying, and other processing procedures. NR exhibits exceptional comprehensive performance in medicine, aviation, tires, and vibration-absorptive material, significantly outperforming synthetic rubbers [1]. However, NR undergoes storage hardening during storage. Storage hardening is a dynamic change that induces structural modification in NR. Ejolle et al. [2] classified the structure of NR into macrostructure, mesostructure, microstructure, and nanostructure. Macrostructure enables researchers to predict the behavior of NR during its processing, such as Mooney viscosity (ML) and Wallace plasticity (P_0_) [3]. Mesostructure and microstructure include macrogel, microgel_>1μ_, microgel_<1μ_, the interactions between polyisoprene chain and non-isoprene compound, molecular scale, the number of branches per chain, and molecular mass [4,5]. Nanostructure refers to the terminal groups of molecular chains, side-chain groups, and group ordering. Storage hardening is characterized by rising ML, P_0_, and macrogel content [6]. The constant viscosity natural rubber (CV), a popular damping material by automobile and high-speed railway, was prepared by adding hydroxylamine reagent. The CV can keep ML, P_0_, and macrogel content constant because the constant viscosity inhibited the cross-linking reactions of the rubber molecular chains.

There are two widely accepted mechanisms for storage hardening of NR. One is that of Sekhar et al. [7,8], who proposed the cross-linking hypothesis of aldehyde groups on the molecular chain of NR. The other is that of Tanaka et al. [9,10], who proposed the cross-linking between the molecular chain terminal groups of NR and non-isoprene compound through hydrogen and ionic bond. Currently, the common constant viscosity technology of NR is required the addition of constant viscosity reagent into the NR to inhibit the reactive behavior of aldehyde groups present on its molecular chains or remove long-chain fatty acids from a phospholipid. There are mainly two preparation techniques for the CV at present. One is to add the constant viscosity reagent prior to the coagulation of fresh latex, which was designated as “latex-mixing method”. The other is to introduce the constant viscosity reagent during the dry-mixing stage in the processing of NR, which is referred to as “dry-mixing method”. Sakdapipanich et al. [11] prepared samples with different constant viscosity reagents by latex-mixing method. They also studied the effect of the phenol and hydroxylamine hydrochloride on the ML, P_0_, and macrogel of the CV under accelerated storage. Promhuad et al. [12] prepared the CV with hydroxylamine sulfate as the constant viscosity reagent by latex-mixing method, and researched the influence of the constant viscosity reagent on ML and macrogel. Chollakup et al. [13] prepared the CV with hydroxylamine hydrochloride as the constant viscosity reagent by latex-mixing method, and studied the effect of the constant viscosity reagent on ML and molecular mass. The researchers focused on the influence of the types of the constant viscosity reagent on the properties or the structure of the CV. Moreover, the samples of these researches were all prepared by latex-mixing method rather than dry-mixing method, because the dry-mixing method is difficult to operate in the laboratory and requires engineering equipment.

In addition, the relationship between macrostructure and micro-mesostructure should be attracted more attention for the study of NR materials rather than single characterization analysis [14,15,16,17,18]. Hayeemasae et al. [14] studied the relationship between the structure and properties of different grades of NR. They found the molecular mass significantly affected ML, torque during processing, and thermo-mechanical and mechanical properties of NR. Saiwari et al. [16] studied the relationship between the structure and properties for different NR clones. They found the molecular mass was positively correlated with P_0_, and that the weight average molecular mass (*M*_w_) affected the radius of gyration and the hydrodynamic radius of NR.

In this paper, we overcame the operation difficulties of dry-mixing method. The CV was prepared by two constant viscosity treatment methods, namely the dry-mixing method and the latex-mixing method. ML, P_0_, macrogel, and microgel_>1μ_ were measured. A gel permeation chromatography (GPC) with refractive index (RI) detector, intrinsic viscometer (IV) detector, and light scattering (LS) detector was used to analyze the microstructure of NR. The impact of the two methods on the mesostructure and microstructure of NR after 48 h accelerated storage was analyzed.

## 2. Materials and Methods

### 2.1. Materials

Fresh latex was provided by Guangdong Guang ken Rubber Group Co., Ltd. (Guangzhou, China) Neutral Hydroxylamine Sulphate (NHS), formic acid, and acetone were analytical grade. Tetrahydrofuran (THF) purchased from J.T. Baker (Phillipsburg, NJ, USA) was high performance liquid chromatography grade. The standard samples of polystyrene (PS) and linear poly(cis-1,4-isoprene) (PI) were supplied by Polymer Standard Service (PSS) in Germany (Mainz, Germany).

### 2.2. Sample Preparation

#### 2.2.1. Preparation of Unaccelerated Storage Samples

**Figure 1:**
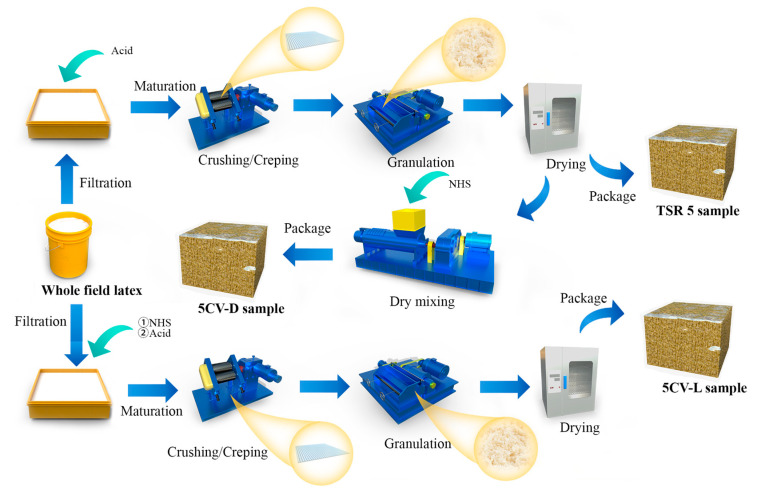
Schematic diagram of preparation process of 5CV-D, 5CV-L, and TSR 5.

The preparation process of the samples designated 5CV-D, 5CV-L, and TSR 5 is shown in Figure 1. TSR 5 was the control sample.

The fresh latex was coagulated with acid. The coagulum was matured for 7 d and subsequently dried at 83 °C to obtain the rubber block. The CV sample was obtained by the dry-mixing method together with 0.15% NSH. labelled 5CV-D.

The fresh latex with 0.15% NHS was coagulated with acid. The coagulum was matured for 7 d and subsequently dried at 83 °C to obtain the CV sample labelled 5CV-L.

The fresh latex was coagulated with acid. The coagulum was matured for 7 d, then dried at 83 °C to obtain the raw rubber control sample labelled TSR 5.

#### 2.2.2. Preparation of Accelerated Storage Samples

The unaccelerated storage samples were homogenized and placed in a dryer filled with phosphorus pentoxide (P_2_O_5_, McLean Company, Palm Springs, CA, USA), then vacuumed for 20 min. The vacuum dryer containing samples was placed in an oven at 60 °C (GZX-9240MBE, Shanghai Boxun Industrial Co., Ltd. Medical Equipment Factory, Shanghai, China) for 48 h. The samples measuring mesostructure and microstructure did not require homogenization.

### 2.3. Testing and Characterization

#### 2.3.1. Determination of Mooney Viscosity and P_0_

ML was measured according to ISO 289-1 [19]. The homogenized sample was cut by the cutting machine with a circular cutter to obtain the testing sample. A group of testing samples consisted of two circular films with a diameter of about 50 mm and a thickness of about 6 mm. ML was tested by using a Mooney viscometer (MV-3000, Dongguan High-Speed Railway Testing Instrument Company, Guangdong, China) with the upper and lower die temperatures at 100 ± 0.5 °C and a large rotor [20]. P_0_ was measured by a Wallace rapid plasticity meter (P14, Wallace Company, London, UK) according to ISO 2007 [21].

#### 2.3.2. Determination of Mesostructure and Microstructure

The testing of mesostructure and microstructure was carried out by using a GPC with refractive index (RI) detector, intrinsic viscometer (IV) detector, and light scattering (LS) detector.

The 0.1 g sample was dissolved in 30 mL THF, then was allowed to stand undisturbed in a dark environment for 24 h. Thereafter, the sample was centrifuged at 14,000 r/h for 2 h using a high-speed frozen centrifuge (CR21N, Hitachi, Tokyo, Japan). The bottom sediment (the insoluble part of NR) defined as “macrogel” was transferred to a dry and clean container. Then, it was dried in an oven at 105 °C until a constant weight was achieved to determine the macrogel content.

The supernatant (the dissolved part of NR) was filtered through the PTFE membrane filter with a porosity of 1 μ and injected in the GPC. The gel remaining on the PTFE membrane filter was designated as “microgel_>1μ_”. The determination of microgel_>1μ_ content was conducted in accordance with the method in the literature [3,6]. The filtrate was analyzed by a triple-detector GPC (1260 II, Agilent, Santa Clara, CA, USA) with a flow rate of 0.65 mL/min and a detection temperature of 40 °C.

## 3. Results and Discussion

**Figure 2:**
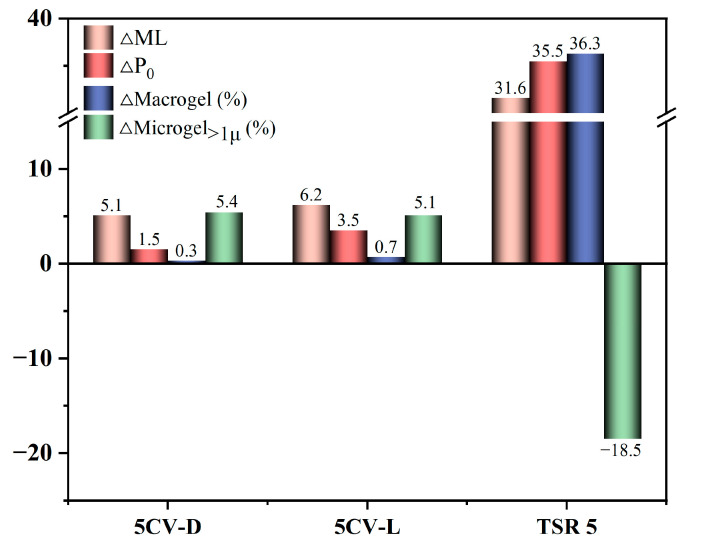
Difference of ML, P_0_, macrogel, and microgel_>1μ_ between accelerated storage and unaccelerated storage.

Table 1 shows ML, P_0_, macrogel, and microgel_>1μ_ for unaccelerated storage and accelerated storage for 48 h. Figure 2 shows the difference of ML, P_0_, macrogel, and microgel_>1μ_ between accelerated storage for 48 h and unaccelerated storage. As shown in Table 1 and Figure 2, ML and P_0_ of 5CV-D and 5CV-L increased slightly after 48 h of accelerated storage, while those of TSR 5 showed a significant increase. This is attributed to the fact that the addition of constant viscosity reagent inhibits the cross-linking reactions of the rubber molecular chains. In addition, the CV prepared by the dry-mixing method exhibits superior constant viscosity performance, characterized by lower ∆ML and ∆P_0_.

From Figure 2, it can be observed that TSR 5 without constant viscosity reagent could not keep the macrogel and microgel_>1μ_ constant during accelerated storage. The macrogel content increased significantly after accelerated storage, while the microgel_>1μ_ content decreased significantly. This is consistent with the results of the literature [22,23,24]. It may be that abnormal groups on short molecular chains interact with the microgel_>1μ_, causing microgel_>1μ_ to form macrogel [25]. The macrogel content of 5CV-D and 5CV-L remains relatively stable during accelerated storage, and the microgel_>1μ_ content of 5CV-D and 5CV-L exhibits a slight increase. This shows the addition of constant viscosity reagent was able to stabilize the macrogel content, but unable to maintain the stability of microgel_>1μ_ content. Bonfils et al. [6,22] have proposed that there are microgel_<1μ_ and many short molecular chains in NR, and the microgel_<1μ_ can crosslink with short molecular chains to form larger gel structures (microgel_>1μ_ and macrogel). Therefore, we infer that the crosslink cannot be stop by the addition of constant viscosity reagent. The abnormal groups of microgel_>1μ_ was controlled, which can make the macrogel structure fail to form by condensation reactions during accelerated storage. But the abnormal groups in the internal microgel_<1μ_ were unable to fully react with the constant viscosity reagent, which made the cross-linking reaction continue to occur and resulted in the formation of the microgel_>1μ_. This indicates that a cross-linking reaction continues to occur within the internal structure of the CV during accelerated storage.

Meanwhile, a comprehensive analysis of measured data for the CV in Figure 2 shows that 5CV-D has a lower ∆ML, ∆P_0_, and ∆ Macrogel. This means that the CV prepared by dry-mixing method had a better constant viscosity effect on these indicators. It may be that there is a similar shearing effect in the dry-mixing process as in the mastication process [4], which exposes more abnormal groups on long molecular chains. Hence, a greater number of abnormal groups were inhibited by the constant viscosity agent. The hardening phenomenon was slowed down.

**Figure 3:**
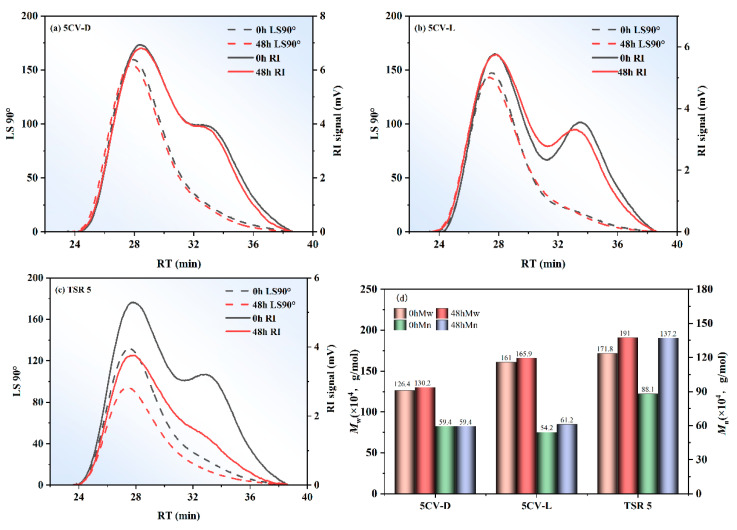
Absolute molecular mass results analysis. (**a**–**c**) Refractometer (RI) signals and light scattering (LS, 90°) signals as a function of elution time of three samples; black indicates unaccelerated storage, red indicates accelerated storage for 48 h. (**d**) Absolute *M*_w_ and absolute *M*_n_ for three samples.

Table 2 shows the absolute molecular mass data of samples unaccelerated storage and after 48 h of accelerated storage. It can be found that the molecular mass of TSR 5 increases significantly after accelerated storage, while the molecular mass of 5CV-L and 5CV-D almost unchanged. It may be that the constant viscosity reagent inhibits the cross-linking of abnormal groups on short molecular chains to form long molecular chains. Moreover, the absolute *M*_w_ of 5CV-L was higher than the absolute *M*_w_ of 5CV-D. This phenomenon may be due to the dry-mixing process, which breaks some molecular chains in NR. As shown in Figure 3, TSR 5 showed clear bimodal molecular weight distribution (MWD) before accelerated storage, and changed to unimodal distribution after accelerated storage. 5CV-L displayed clear bimodal MWD, while 5CV-D exhibited unimodal MWD with a minor shoulder. Meanwhile, it can be found that the MWD of 5CV-D and 5CV-L remained unchanged after accelerated storage. This means that the constant viscosity reagent had taken effect, and the polydispersity index (*PDI*) is related to the MWD. Table 2 and Figure 3 show the sample with bimodal MWD had a high *PDI*, while the sample with unimodal MWD had a low *PDI*. When the MWD remained consistent, the PDI also exhibited minimal variation. For the stability of absolute molecular mass and MWD, both methods achieved satisfactory constant viscosity performance when preparing the CV.

**Figure 4:**
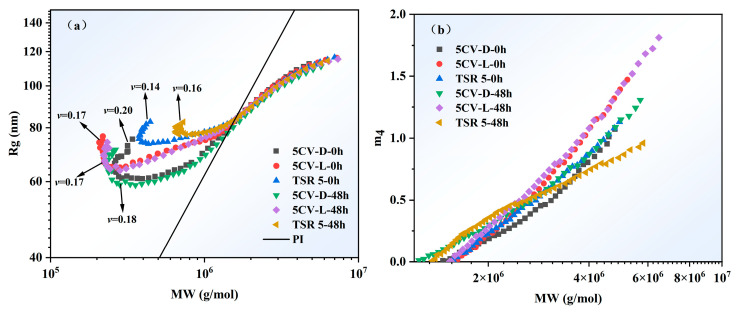
(**a**) Conformation plot of three samples; straight line is conformation plot of standard linear PI with a Flory exponent (*ν*) of 0.654 from reference [30]. (**b**) Distributions of number of branches per chain (*m*_4_) of three samples as a function of molecular mass.

The radius of gyration (*R*g) refers to the root mean square distance from all atoms in a molecule to the center of mass, which was used to describe the curl degree and the molecular scale of polymer. The slope (Flory exponent, *ν*) for the *R*g as a function of the absolute *M*_w_ of three samples can be utilized to obtain information about the shape of polymer chains [26,27]. The values of *ν* were 0.14 to 0.20 in Figure 4a, which was much lower than the theoretical values of 0.5 to 0.6 (random coil). This means that the conformation of NR molecule was special. The polyisoprene molecular chain in THF may be compact spherical structure or highly branched molecule [28]. The molecular chains shape of NR would not change with the addition of constant viscosity reagent, nor would it change with the dry-mixing process. The low molecular mass position showed abnormal elution (upsweep). This phenomenon was caused by the co-elution of short molecular chains (low molecular mass) and microgel_<1μ_ [29]. The degree of the abnormal elution would be used to qualitatively analyze microgel_<1μ_ content. A high degree of abnormal elution indicates a high content of microgel_<1μ_ [29,30,31]. Hence, it can be roughly estimated that the microgel_<1μ_ content of 5CV-D is low, while the microgel_<1μ_ content is high. And the quantitative analysis of microgel_<1μ_ content will be investigated in greater depth in the future.

The branched points of the molecular chains were assumed to be tetra-functional. Figure 4b presents the distributions of the number of branches per chain (*m*_4_) of three samples as a function of molecular mass, with standard linear poly(cis-1,4-isoprene) (PI) used as a reference. The *m*_4_ values of three samples increased proportionally with the molecular mass. The values of *m*_4_ for 5CV-L stayed almost stable after accelerated storage, while the values of *m*_4_ for 5CV-D slightly increased. The *m*_4_ values of the low molecular mass position of TSR 5 increased after accelerated storage, while those of the high molecular mass position decreased. The maximum value of *m*_4_ for 5CV-L was approximately 1.81, whereas that for 5CV-D was about 1.31. This difference may be attributed to the disruption of highly branched molecular chains caused by dry-mixing process. Hence, the latex-mixing method for preparing the CV showed better advantage in protecting *m*_4_.

## 4. Conclusions

In this study, the CV was prepared by two constant viscosity treatment methods. The GPC with triple detector was used to measure the microstructure of the sample. The dynamic changes of macrostructure, mesostructure, and microstructure of the sample after accelerated storage for 48 h were comprehensively discussed. It was found that after 48 h of accelerated storage, ML and P_0_ of TSR 5 without constant viscosity reagent increased rapidly, while ML and P_0_ of the CV prepared by latex-mixing method and dry-mixing method respectively were controlled to a certain extent. The CV prepared by the dry-mixing method exhibited superior constant viscosity performance during accelerated storage, as evidenced by lower ∆ML and ∆P_0_.

TSR 5 without constant viscosity reagent cannot keep the macrogel and microgel_>1μ_ constant during accelerated storage. The macrogel content increased significantly after accelerated storage, and the microgel_>1μ_ content decreased significantly after accelerated storage. This might be attributed to the interaction between the abnormal groups on the short molecular chains and the microgel_>1μ_, resulting in the formation of macrogel from the microgel_>1μ_. The CV prepared by the two constant viscosity treatment methods can maintain the stability of the macrogel structure but failed to preserve microgel_>1μ_ in structure. It was inferred that the addition of constant viscosity reagent controlled abnormal groups with microgel_>1μ_ and macrogel, but the internal microgel_<1μ_ could not be controlled. This caused the microgel_<1μ_ to continue to cross-linking with the short molecular chains to form microgel_>1μ_.

For the stability of MWD, the absolute *M*_n_ and *M*_w_, the MWD of NR without constant viscosity reagent changed from bimodal to unimodal after accelerated storage, and the molecular mass could not be maintained. In addition, both CV prepared via the two methods exhibited satisfactory stability in MWD and absolute molecular mass during accelerated storage. However, the drying-mixing process disrupts molecular chains, resulting in low absolute *M*_w_. The sample with bimodal MWD corresponded to a high *PDI*, while the sample with unimodal MWD corresponded to a low *PDI*. The polyisoprene molecular chains of NR in THF displayed compact spherical structure or highly branched molecule, as reflected by the *ν* values (0.14 to 0.20), which remained unaffected by the addition of constant viscosity reagent or the dry-mixing process. The CV prepared by the latex-mixing method demonstrated higher *m*_4_. Hence, the influence of the two CV preparation methods during accelerated storage on the structure should be comprehensively evaluated from different structures.

Therefore, the effects of the two preparation techniques for the CV on the structure and the constant viscosity effect during storage should be evaluated comprehensively from the macrostructure and micro-mesostructure. It can provide more theoretical support for the sample preparation and research of the CV with stable storage performance. In addition, the dynamic evolution in microgels_<1μ_, short molecular chains, and other microstructural features in NR are also worthy of future research, which needs to explore more advanced characterization.

## Figures and Tables

**Table 1:** Macrostructure and mesotructure of unaccelerated storage and accelerated storage for 48 h.

Sample	ML		P_0_		Macrogel	
	0 h	48 h	0 h	48 h	0 h	48 h
5CV-D	67.7	72.8	34.0	35.5	10.8	11.1
5CV-L	67.4	73.6	33.0	36.5	15.0	15.7
TSR 5	83.6	115.2	45.5	81.0	19.5	55.8

**Table 2:** Absolute *M*_w_, the absolute *M*_n_, and *PDI* of unaccelerated storage and accelerated storage for 48 h.

Sample	*M*_w_ (g/mol), ×10^4^		*M*_n_ (g/mol), ×10^4^		*PDI*	
	0 h	48 h	0 h	48 h	0 h	48 h
5CV-D	126.4	130.2	59.4	59.4	2.1	2.2
5CV-L	161.0	165.9	54.2	61.2	3.0	2.7
TSR 5	171.8	191.0	88.1	137.3	2.0	1.4

## Data Availability

The original contributions presented in this study are included in the article. Further inquiries can be directed to the corresponding authors.
